# Moyamoya disease in a Moroccan baby: a case report

**DOI:** 10.1186/s13256-018-1642-y

**Published:** 2018-06-13

**Authors:** Abdelhafid Houba, Nisrine Laaribi, Mohammed Meziane, Abdelhamid Jaafari, Khalil Abouelalaa, Mustapha Bensghir

**Affiliations:** 1 Department of Anesthesiology, Military Hospital Mohammed V Rabat, Faculty of Medicine and Pharmacy, University of Mohammed V, Souissi, district Riyadh, BP: 1000 Rabat, Morocco; 2 Department of Pediatric, Children’s Hospital Rabat, Faculty of Medicine and Pharmacy, University of Mohammed V, Souissi, Rabat, Morocco

**Keywords:** Acute stroke, Moyamoya disease, Cerebral angiography, Infant

## Abstract

**Background:**

A stroke in a baby is uncommon, recent studies suggested that their incidence is rising. Moyamoya disease is one of the leading causes of stroke in babies. This condition is mostly described in Japan. In Morocco, moyamoya disease has rarely been reported and a few cases were published. We report a rare Moroccan case of a 23-month-old baby boy who presented with left-sided hemiparesis and was diagnosed as having moyamoya disease.

**Case presentation:**

A 23-month-old full-term Moroccan baby boy born to a non-consanguineous couple was referred to our hospital with the complaint of sudden onset left-sided hemiparesis. On neurological examination, there were no signs of meningeal irritation, his gait was hemiplegic, tone was decreased over left side, power was 2/5 over left upper and lower limb, and deep tendon reflexes were exaggerated. Preliminary neuroimaging suggested an arterial ischemic process. Clinical and laboratory evaluation excluded hematologic, metabolic, and vasculitic causes. Cerebral angiography confirmed the diagnosis of moyamoya disease. Our patient was treated with acetylsalicylic acid 5 mg/kg per day and referred to follow-up with pediatric neurosurgeon. Cerebral revascularization surgery using encephaloduroarteriosynangiosis was performed. At 8-month follow-up, his hemiparesis had improved and no further ischemic events had occurred.

**Conclusion:**

This case highlights the importance of considering moyamoya disease to be one of the classic etiologies of acute ischemic strokes in children from North Africa. It also emphasizes the rare presentation among the African population and the use of neurovascular imaging techniques to facilitate diagnosis of moyamoya disease.

## Background

Moyamoya disease (MMD) is a chronic cerebrovasculopathy of unknown etiology that is characterized by progressive steno-occlusive lesions in the supraclinoid internal carotid artery and/or its main branches in the circle of Willis. To compensate for the blood flow around the occlusive region, a fine vascular network develops that resembles “puffs of smoke”, thus, the Japanese term “moyamoya” [1, 2]. The unique appearance of moyamoya vessels described by Suzuki and Takaku in 1969 spurred international recognition of MMD [3].

MMD was originally considered exclusive to East Asia, with particular prevalence in Japan, but it is now increasingly diagnosed throughout the world, and represents an important cause of childhood stroke [4, 5]. In Morocco, MMD has rarely been reported and a few cases were published. In the present report, we describe a rare Moroccan case of a 23-month-old baby boy who presented with acute left-sided hemiparesis diagnosed as MMD.

## Case presentation

**Fig. 1:**
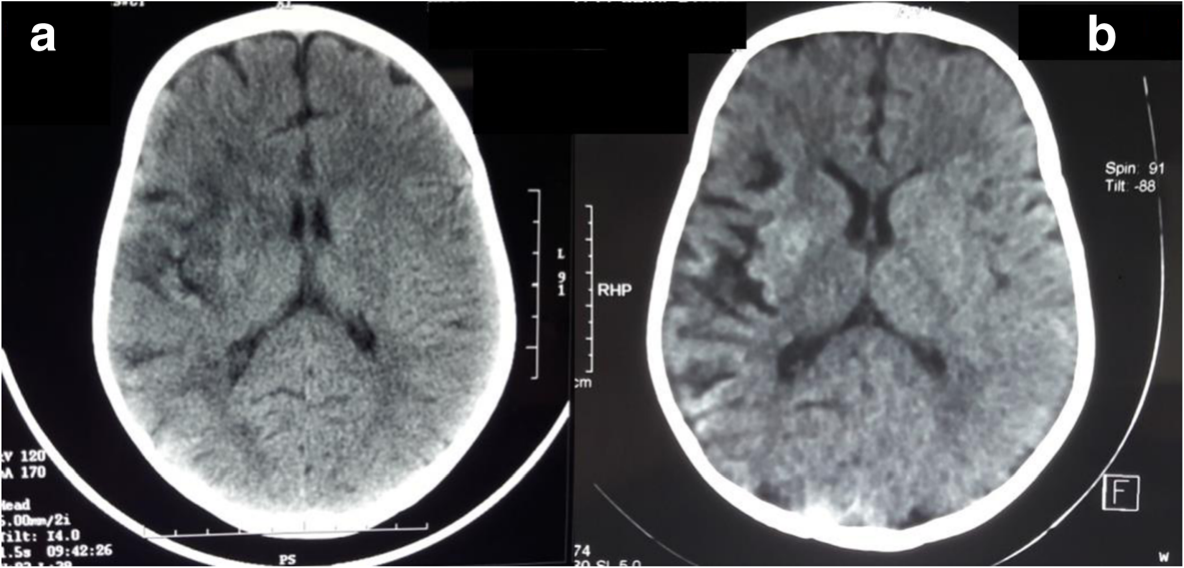
Brain computed tomography showing, at day 3 of deficit, a low density area in the right cortical and subcortical frontotemporoparietal regions and right insular cortex representing ischemic infarcts in the territory of the right middle cerebral artery (**a**). Evolution of previous ischemic lesion at day 9 of deficit (**b**)

A 23-month-old full-term Moroccan baby boy born to a non-consanguineous couple was referred to our hospital with the complaint of sudden onset left-sided hemiparesis evolving for 2 days. There was no history of fever, seizure, head injury, ear discharge, headache, visual disturbances, or vomiting. No family history of early stroke, ischemic heart disease, or autoimmune disease was reported. At presentation, he was conscious and well orientated. There was no dysmorphism, no neurocutaneous markers, and he had normal growth parameters. He was afebrile with a pulse of 110 beats per minute and a respiratory rate of 26 breaths per minute. On neurological examination, there were no signs of meningeal irritation, his gait was hemiplegic, tone was decreased over left side, power was 2/5 over left upper and lower limb using Medical Research Council (MRC) muscle strength scale, and deep tendon reflexes were exaggerated. There were no abnormalities on cardiac auscultation or hepatosplenomegaly. Brain computed tomography (CT) showed a low density area in the right cortical and subcortical frontotemporoparietal regions and right insular cortex representing ischemic lesions in the right middle cerebral artery (MCA) territory (Fig. 1). Laboratory investigations, including hemogram, prothrombin, partial thromboplastin, erythrocyte sedimentation rate (ESR), fibrinogen, proteins C and S, antithrombin III activity, and antinuclear antibody tests were all within limits. Renal ultrasonography with Doppler flow study showed no evidence of renal artery stenosis. Electroencephalography revealed no epileptic discharges, only a diffuse slow rhythm over the left hemisphere with slow waves at the temporal regions.

**Fig. 2:**
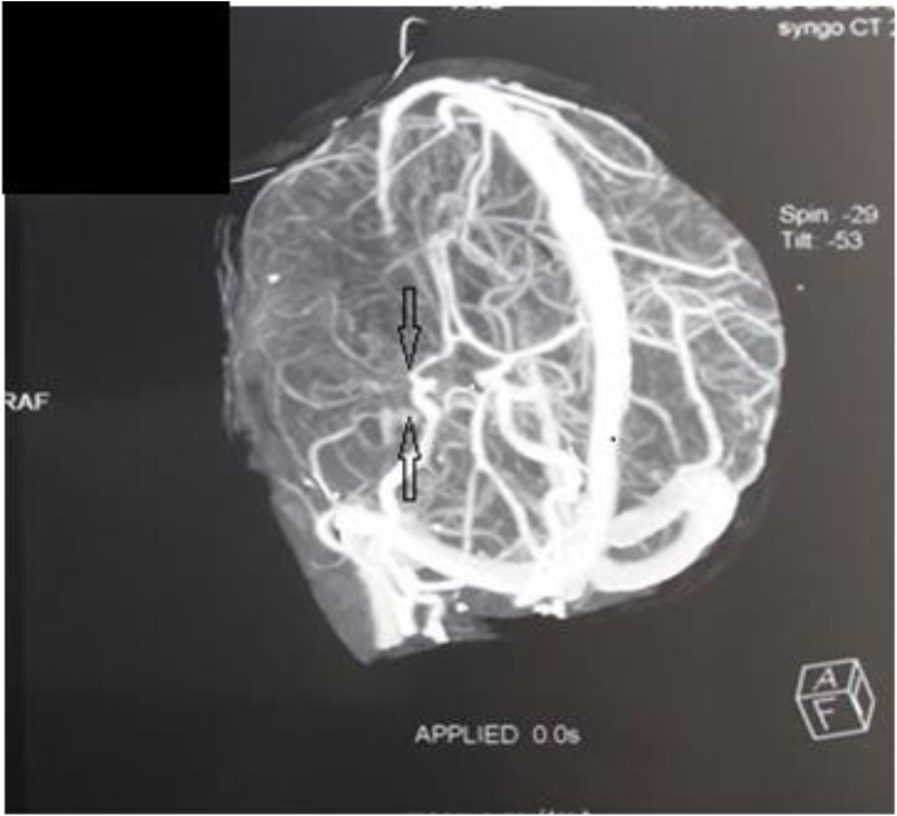
Cerebral angiography at the level of the circle of Willis showed a proximal obliteration (arrows) in the right middle cerebral artery with a fine anastomotic Moyamoya network and typical “puff of smoke” appearance

Our patient was booked for cerebral angiography with contrast, which revealed a proximal obliteration in his MCA with a fine anastomotic moyamoya network and typical “puff of smoke” appearance (Fig. 2). Urine for metabolic screening, including homocysteine levels, was normal. A normal hemoglobin electrophoresis ruled out sickle cell disease. An electrocardiogram and echocardiogram revealed no abnormalities. He was finally diagnosed as a case of MMD.

We treated our patient conservatively with acetylsalicylic acid 5 mg/kg per day. He showed slight improvement in power over left side (3/5). He was then discharged to follow-up with pediatric neurosurgeon. Cerebral revascularization surgery using encephaloduroarteriosynangiosis (EDAS) was performed. At 8-month follow-up, his hemiparesis was improved and no further ischemic events had occurred.

## Discussion

Compared with adults, acute stroke is an infrequent disease of pediatric patients. In fact, the reported incidence of childhood stroke has increased in the last 20 years according to population studies, most likely related to improvements in neuroimaging techniques [6]. Updated incidence figures from the Canadian Pediatric Ischemic Stroke Registry showed that ischemic stroke in childhood occurs in 3.3 per 100,000 children per year [7].

There are numerous causes of stroke in childhood, classified in major categories as shown in Table 1 [5, 7]. These include congenital heart disease, sickle cell disease, immune disorders, clotting disorders, and head and neck trauma. However, MMD should be included in the differential for a stroke in a child. In fact, MMD represents one of the most severe arteriopathies and accounts for approximately one fifth of identified cerebral arteriopathies in children presenting with acute stroke and is diagnosed in up to 20% of cases of childhood acute ischemic stroke (AIS) [8]. The condition is common in Japan and East Asia, where familial cases are also clearly recognized. In Japan, the annual prevalence and incidence have been estimated to be 3.16–10.5 and 0.35–0.94 per 100,000 [9]. The female to male ratio has been shown to be 1.8-2.2 (female predominance) [10]. The familial form accounts for 10–15% [11].

**Table 1:** Causes for pediatric acute ischemic stroke classified in major categories

Arteriopathies	Arterial fibromuscular dysplasia, arteriovenous malformation, arterial dissection, moyamoya disease, transient cerebral arteriopathy of childhood, primary central nervous system vasculitis, cranial radiotherapy
Vasculitis	Meningitis, postinfectious systemic lupus erythematosus, polyarteritis nodosa, granulomatous angiitis, Takayasu’s arteritis, rheumatoid arthritis, dermatomyositis, inflammatory bowel disease, hemolytic uremic syndrome
Hematologic disorders and coagulopathies	Hemoglobinopathies (sickle cell anemia, sickle cell–hemoglobin C, sickle-thalassemia), purpura, thrombocytosis, polycythemia, disseminated intravascular coagulation, leukemia or other neoplasms, congenital coagulation defects, lupus anticoagulant, anticardiolipin antibodies
Metabolic disorders	Mitochondrial disorders (MELAS syndrome), urea metabolic disorders, homocystinuria, aminoaciduria, glutaric acidemia type I, lysosomal disorders, Fabry’s disease
Heart diseases	Congenital malformations: ventricular/atrial septal defect, patent ductus arteriosus, aortic/mitral stenosis, coarctation, complex congenital heart defects Acquired: rheumatic heart disease, endocarditis, myocarditis, arrhythmia
Traumatic	Child abuse, post-traumatic arterial dissection, blunt cervical arterial trauma, post-traumatic carotid cavernous fistula, penetrating intracranial trauma

By Rosa *et al.* [29] (licensed under a CCBY4.0 licence: https://creativecommons.org/licenses/by/4.0/)

*MELAS* mitochondrial myopathy, encephalopathy, lactic acidosis, and stroke-like episodes

The etiology of MMD remains unknown, but recent advances have been made in understanding the molecular biology and physiopathology of this rare entity. Previous studies explored genetic factors and revealed several loci associated with MMD: 3p24-p26, 6q25, 8q23, and 17q25. More recently, the *RNF213* gene in the 17q25-ter region was identified as a novel susceptibility gene for MMD among East Asian population. Mutational analysis of *RNF213* revealed a founder mutation, p.R4859K, in 95% of families with MMD, 73% of cases of non-familial MMD, and 1.4% of the control cases. A polymorphism in c.14576G>A in *RNF213* was identified in 95% of familial patients with MMD and 79% of sporadic cases, and *RNF213* was found to correlate with the early onset and severe forms of MMD, indicating its value as a good biomarker for predicting prognosis [12]. Also, Xq28 deletions removing *MTCP1*/*MTCP1NB* and *BRCC3* have been shown to cause a type of X-linked familial moyamoya syndrome [13].

MMD is rarely seen in the African population. Uchino *et al*. found only 27 African Americans with MMD in the states of California and Washington [14]. In this observation, the diagnosis of the disease of moyamoya was retained on the basis of angiographic findings in a Moroccan 23-month-old baby who presented with left-sided hemiparesis.

Moyamoya is categorized as MMD when there is no underlying etiology or association and as moyamoya syndrome if an underlying etiology or association of other conditions is recognized, including trisomy 21, Down syndrome, neurofibromatosis type 1, and cranial irradiation. The main conditions associated with moyamoya syndrome are summarized in Table 2 [15, 16]. In our patient, no association of any other systemic disorder was described. Furthermore, most general medical conditions were excluded by normal blood, urine studies, and negative history of fever or trauma. A normal physical examination ruled out increased intracranial pressure as well as meningitis.

**Table 2:** Conditions reported in association with moyamoya syndrome

More common	Less common
Neurofibromatosis type I	Structural cardiac anomalies
Sickle cell disease	PHACES syndrome
Down syndrome (trisomy 21)	Congenital dwarfing syndromes
Post-cranial radiation	Alagille syndrome

Permission was granted by Kirkham and deBaun (© Current Science Inc. [30]) to reuse this table

*PHACES* posterior fossa malformations–hemangiomas–arterial anomalies–cardiac defects–eye abnormalities–sternal cleft and supraumbilical raphe syndrome

A bimodal age distribution has been reported for MMD: a high peak at 5 years and a low peak at approximately 40 years [5]. A recent report, however, revealed a highest peak between 45 and 49 years followed by a second peak between 5 and 9 years [17]. This suggests that the incidence of pediatric MMD has started to decrease [17].

Pediatric MMD usually presents with cerebral ischemia (80%) rather than hemorrhage (20%) [18]. Immature verbal skills in younger children make it difficult for early diagnosis (transient ischemic attacks versus infarction) [19]. Ischemic events can occur secondary to crying, blowing, or hyperventilation. This behavior induces hypocapnic vasoconstriction or vasospasm in an already compromised cerebral circulation [20]. Patients can also present with headaches, seizures, involuntary movements, and progressive decline in intellectual ability [21].

The diagnosis of MMD is based on the characteristic angiographic appearance of stenosis affecting the distal internal carotid artery and/or proximal circle of Willis’s vessels, along with the presence of prominent basal collateral vessels. The disease tends to be progressive and it can be unilateral or bilateral. Definitive diagnosis requires neurovascular imaging such as catheter angiogram, CT angiogram, and magnetic resonance angiogram (MRA). A brain CT is typically the first study obtained which shows areas of hypodensity consistent with infarction, and less commonly hemorrhages or atrophy [22]. It showed in our case a low density area in the right cortical and subcortical temporoparietal regions and right insular cortex representing ischemic infarcts in the territory of the right MCA. MRA is very useful for diagnosing MMD with sensitivity of 73% and specificity of 100% [23]. Sensitivity increases to 92% when MRA is combined with magnetic resonance (MR) imaging [24]. However, the smaller moyamoya collaterals are visualized more clearly with conventional cerebral angiography that is still the gold standard for diagnosing and surgical decision making for MMD [22, 25, 26].

Although there has not been any randomized controlled trial comparing surgical and medical treatment in patients with MMD, surgical revascularization has been accepted as the only effective form of treatment. Multiple case series, both retrospective and prospective, have shown the effectiveness of revascularization procedures in preventing future ischemic episodes in patients with MMD [13, 27]. Medical management may involve drugs such as antiplatelet agents which are generally given to prevent thrombosis. Surgical procedures are classified into three categories: direct bypassing including superficial temporal artery (STA) to MCA anastomosis, indirect bypassing including EDAS and encephalomyosynangiosis (EMS), and combined bypassing. Direct bypassing can be technically challenging in some pediatric patients with cortical arteries of smaller diameter, but can improve cerebral hemodynamics immediately after surgery. Indirect bypass surgery that induces spontaneous angiogenesis between the brain surface and the vascularized donor tissues is technically simple, but requires 3–4 months for the collaterals to develop. Prognosis of patients with MMD is found to be related to age and the type of presentation [13]. In a Japanese study, EDAS was proved to be an efficacious procedure benefitting 75% of patients suffering from transient ischemic attacks within 1 year [28].

## Conclusions

This case highlights the importance of considering MMD to be one of the main etiologies of AIS in children from North Africa. It also emphasizes the rare presentation among the African population and the use of neurovascular imaging techniques to facilitate diagnosis of MMD.

## Abbreviations

AIS: Acute ischemic stroke
CT: Computed tomography
EDAS: Encephaloduroarteriosynangiosis
EMS: Encephalomyosynangiosis
ESR: Erythrocyte sedimentation rate
MCA: Middle cerebral artery
MMD: Moyamoya disease
MR: Magnetic resonance
MRA: Magnetic resonance angiogram
MRC: Medical Research Council
STA: Superficial temporal artery
